# Asymptomatic Abnormalities in the Knee, Shoulder, and Ankle Joints of Collegiate Athletes: A Cross-Sectional MRI-Based Comparative Study

**DOI:** 10.3390/diagnostics16091335

**Published:** 2026-04-29

**Authors:** Na Jiang, Hanqi Wang, Xinyu Zhang, Jia Chen, Gang Wei, Fuhua Yan, Xiaokai Li, Yong Lu

**Affiliations:** 1 Department of Radiology, Ruijin Hospital, Shanghai Jiao Tong University School of Medicine, Shanghai 200025, China; 2 School of Sport Rehabilitation, Shanghai University of Sport, Shanghai 200438, China

**Keywords:** asymptomatic states, magnetic resonance imaging, athletes, sports medicine

## Abstract

**Background**: Asymptomatic structural joint abnormalities are prevalent among athletes, yet studies on their multi-joint distribution and comparisons with low-activity controls remain lacking. This article evaluated the prevalence and characteristics of asymptomatic structural abnormalities across joints in collegiate athletes compared with controls using 3.0-T MRI. **Methods**: The cross-sectional study enrolled 53 asymptomatic elite collegiate athletes (high physical activity, HPA) and 84 healthy volunteers (low physical activity, LPA) aged 18–25 years. All participants were asymptomatic with no history of joint trauma or surgery. Generalized estimating equation (GEE) logistic regression was employed to identify independent risk factors for joint abnormalities after evaluation. **Results**: A total of 666 joints were analyzed. Participants with at least one joint abnormality were significantly more common in the HPA group than LPA group (49.1% vs. 6.0%, *p* < 0.001). At the joint level, overall abnormality prevalence was 13.5% versus 2.2%, respectively. In the HPA group, knee joints were the most frequently affected (24.2%), predominantly involving meniscal lesions. Shoulder pathologies consisted exclusively of supraspinatus tendon lesions (6.8%), while ankle abnormalities were primarily bone marrow edema (5.9%). GEE analysis identified high physical activity (adjusted OR = 5.23; 95% CI: 1.55–17.71; *p* = 0.008) and elevated BMI (adjusted OR = 1.09 per kg/m^2^; 95% CI: 1.03–1.15; *p* = 0.001) as independent risk factors. **Conclusions**: Asymptomatic abnormalities are highly prevalent and demonstrate intra-individual clustering across multiple joints. MRI-based surveillance represents a promising strategy for early risk identification and injury prevention.

## 1. Introduction

The knee, shoulder, and ankle joints sustain high mechanical stress during athletic activities and are essential for athletes’ competitive performance and long-term sports health. However, athletes face a significantly elevated risk of joint injuries and the risk is notably high across various sports [1,2,3]. Joint injuries in athletes not only impair short-term performance but may also lead to persistent pain, functional limitations, and an increased risk of early-onset osteoarthritis [4,5]. In athletic populations, these three joints are more frequently affected by both acute traumatic injury and chronic overuse pathology [4,5,6].

Athletes aged 18–25 years are in a critical phase of their career: this simultaneously represents the peak window for the development of competitive capacity and performance enhancement, as well as the period of maximal cumulative injury risk. By this stage, most athletes have undergone years of structured sport-specific training, during which the joints have sustained high mechanical loading. Focusing exclusively on symptomatic injuries may overlook the early stages of joint pathologies. Early-stage joint microstructural damage frequently develops silently, preceding the onset of pain or functional decline, and a substantial proportion of such subclinical pathology in athletes remains undetected until clinical manifestation occurs. Prior MRI-based studies have consistently reported high prevalences of asymptomatic structural abnormalities across sports: all Major League Baseball pitchers examined had at least one shoulder abnormality [7], 100% of professional football players showed meniscal or chondral pathologies correlating with training duration [8], and approximately half of amateur ice hockey players had abnormal meniscal or cartilage signals [9].

Nevertheless, the majority of prior studies have been confined to a single sport or a single joint [10,11,12,13] and employed retrospective designs focused on confirmed symptomatic injuries. Furthermore, age-matched non-athletic control groups have rarely been included, making it difficult to distinguish training-related structural adaptations from age-related physiological variation. Magnetic resonance imaging (MRI) has been used as a sensitive tool for detecting musculoskeletal abnormalities. Most studies have relied on conventional two-dimensional MRI sequences with limited spatial resolution, which may underestimate the true prevalence of subtle early structural abnormalities.

To address these gaps, we conducted a cross-sectional MRI-based study employing high-resolution three-dimensional sequences to simultaneously evaluate three major joints (knee, shoulder, and ankle) in collegiate athletes, with a similarly aged low-activity control group for comparison. The objective of this study was to evaluate the prevalence and characteristics of asymptomatic structural abnormalities in collegiate athletes and analyze injury patterns across subgroups stratified by sex, sport type, and training duration.

## 2. Materials and Methods

This cross-sectional, MRI-based comparative study was approved by the Institutional Review Board of Ruijin Hospital, Shanghai Jiao Tong University School of Medicine (RJH-2024-157) on 24 May 2024. Written informed consent was obtained from all participants.

### 2.1. Study Participants

**Figure 1:**
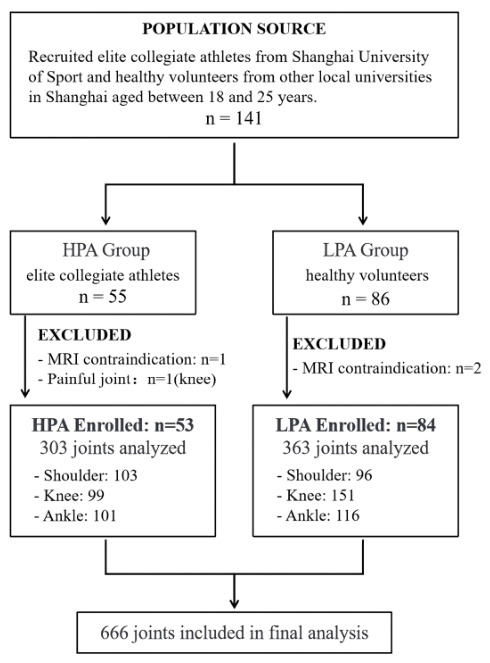
**Participants’ recruitment flowchart.** HPA: high physical activity; LPA: low physical activity.

From September 2024 to June 2025, a total of 137 participants aged between 18 and 25 years were recruited based on their athletic training background: a high-physical-activity (HPA) group of 53 elite collegiate athletes from Shanghai University of Sport, and a low-physical-activity (LPA) group of 84 healthy volunteers from other local non-athletic universities in Shanghai (Figure 1). Self-reported outcome measures (KOOS, ASES, and FAOS) were used to provide a standardized confirmation of asymptomatic status. The exclusion criteria were as follows: (1) MRI scanning contraindications (such as metallic implants, pacemakers, or claustrophobia); (2) a history of joint trauma or surgery involving the joint of interest; and (3) self-reported pain, functional limitations or other clinical symptoms in the scanned joint within the month prior to MRI examination.

We used the Physical Activity Rating Scale-3 (PARS-3) to assess the participants’ physical activity [14]. The PARS-3 evaluates exercise intensity, duration, and frequency and the scores were classified into low (≤19), moderate (20–42), and high (≥43) physical activity levels. HPA group participants were required to have a PARS-3 score ≥ 43, whereas LPA controls were required to have a score ≤ 42.

### 2.2. MRI Examination

All participants rested for at least 30 min before scanning to minimize recent activity-related interference. Participants underwent MRI scanning of the shoulder, knee and/or ankle joints strictly excluding any joints with a history of prior trauma or surgery. For the HPA group, all joints were scanned except those with a history of injury or surgery. For the LPA participants, scanning was limited to 4–6 joints per participant due to time constraints. To ensure technical consistency across both recruiting institutions, all MRI examinations were performed on identical 3.0-T scanners (MAGNETOM Prisma, Siemens Healthineers, Forchheim, Germany), using a 15-channel knee coil, 16-channel shoulder coil, and 20-channel head coil for ankle imaging. The imaging protocol included a 3D T1-weighted SPACE sequence and 3D proton density-weighted fat-suppressed (PD-FS) SPACE sequence. The detailed scan parameters are summarized in Table 1.

### 2.3. Imaging Interpretation

Two experienced musculoskeletal radiologists (H.W. and Y.L., with 8 and 20 years of experience, respectively) independently interpreted all the images. Both reviewers were blinded to the subjects’ information, group allocation and each other’s assessments. If the independent opinions were not in full agreement, a consensus was achieved through discussion. The inter-rater reliability for MRI interpretation was κ = 0.89 (95% CI: 0.71–0.98). We adhered to the definitions, classifications, and methods for recording and reporting of epidemiological data on injury and illness in sports as described in the International Olympic Committee (IOC) consensus statement [15].

The images were evaluated to detect the presence of abnormalities. Specifically, the assessed lesions included: rotator cuff tendon abnormalities in the shoulder; meniscal lesions, ligamentous lesions, cartilage abnormalities, and bone marrow edema in the knee; and Achilles tendinopathy, ligament lesions, and bone marrow edema in the ankle. These abnormalities were defined as follows: bone marrow edema was defined as an ill-defined area of increased signal intensity within the subchondral or cancellous bone on PD-FS images, with corresponding low signal intensity on T1-weighted images [16]. Ligament lesions were defined as abnormal thickening, discontinuity, waviness, or increased signal intensity of the ligament on PD-FS sequences [17]. Cartilage abnormality was defined as focal surface irregularity, partial-thickness or full-thickness cartilage defect, or abnormal signal intensity within the articular cartilage [18]. Achilles tendinopathy was defined as an increased signal on T2-weighted or PD-FS images, tendon thickening, insertional irregularity, or peritendinous edema [19]. The rotator cuff and meniscus lesion grading criteria used in this study are summarized as follows:

Lesions of the rotator cuff were graded according to a modification of the semi-quantitative grading system of Zlatkin et al. [20].

• Grade 0: Normal;
• Grade I: Increased T2-weighted signal with normal morphology;
• Grade II: Increased T2-weighted signal with abnormal morphology (thickening or irregularity of the tendon);
• Grade III: Full-thickness tear (tendon discontinuity).

The degree of meniscus lesions was graded separately according to the system described by Stoller et al. [21]:

• Grade 0: Normal signal;
• Grade I: One or several punctate signal intensities that do not reach the surface of the meniscus;
• Grade II: Linear signal intensity that does not reach the surface of the meniscus;
• Grade III: Signal intensity that reaches the articular surface.

### 2.4. Statistical Analysis

The normality of continuous variables was assessed using the Shapiro–Wilk test. Continuous variables (age, weight, BMI, and PARS-3 scores) were compared using the independent samples *t*-test or the Mann–Whitney U test as appropriate. The Chi-square test or Fisher’s exact test was used to compare gender distribution and the prevalence of joint abnormalities at the patient level between the two groups.

The athletes were categorized into high-impact and low-impact groups based on the predominant mechanical loading characteristics of their sport. High-impact sports are activities that oppose gravity and involve frequent jumping and landing, placing substantial stress on the joints and musculoskeletal system (e.g., running, basketball, soccer, and badminton). In contrast, low-impact sports are activities that minimize jolting and jostling movements, thereby exerting comparatively less mechanical stress on the joints (e.g., swimming, shooting, and cycling) [22].

The intraclass correlation coefficient (ICC) was calculated to quantify the degree of intra-individual correlation among joint-level outcomes, thereby informing the selection of the exchangeable working correlation structure in the GEE models. Two separate generalized estimating equation (GEE) models with a binomial distribution, logit link function, and exchangeable working correlation structure were constructed. The first model evaluated the association between the group (HPA vs. LPA) and the presence of any joint abnormality at the participant level, with sex, age, and BMI included as covariates. The second model evaluated joint-level abnormalities with group, sex, age, BMI, sport type, and training duration included as covariates. Results are reported as adjusted odds ratios (ORs) with 95% confidence intervals (CIs).

Inter-observer reliability was assessed using Cohen’s kappa coefficient. All analyses were performed using R (version 4.4.3; R Foundation for Statistical Computing, Vienna, Austria). A two-sided *p* < 0.05 was considered statistically significant.

## 3. Results

### 3.1. Participant Characteristics

In total, 137 participants were enrolled in the study (53 in the HPA group and 84 in the LPA group), and their characteristics are summarized in Table 2. No significant difference in BMI was observed between groups (*p* = 0.319), whereas the PARS-3 scores were significantly higher in the HPA group compared to the LPA group (*p* < 0.001).

### 3.2. Prevalence of Joint Abnormalities at the Participant Level

The proportion of participants with at least one abnormality was 49.1% (*n* = 26) in the HPA group and 6.0% (*n* = 5) in the LPA group (*p* < 0.001). A total of 303 joints (103 shoulder joints, 99 knee joints, and 101 ankle joints) from the HPA group and 363 joints (96 shoulder joints, 151 knee joints, and 116 ankle joints) from the LPA group were analyzed. Table 3 summarizes the MRI findings of joint abnormalities in the two groups. The overall prevalence of joint abnormality was 13.5% (41/303) in the HPA group and 2.2% (8/363) in the LPA group. After Bonferroni correction, the prevalence of any lesion was significantly higher in the HPA group for shoulder joints (6.8% [7/103] in HPA vs. 0% in LPA; *p* = 0.014), knee joints (24.2% [24/99] vs. 4.6% [7/151]; *p* < 0.001), and ankle joints (9.9% [10/101] vs. 0.9% [1/116]; *p* = 0.003).

**Figure 2:**
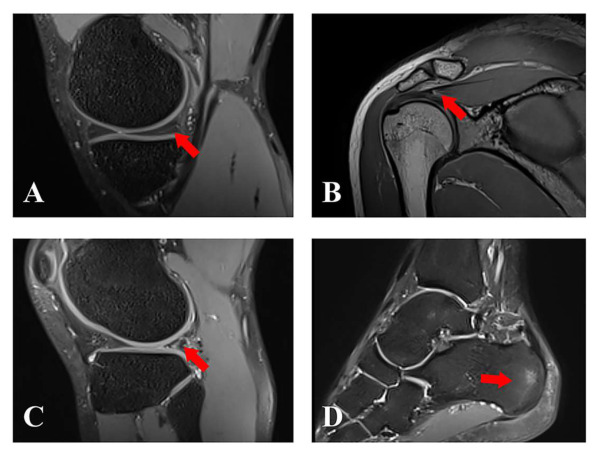
**Representative MRI findings of asymptomatic joint abnormalities in collegiate athletes.** (**A**) Sagittal PD-FS image of the right knee in a 21-year-old male badminton player: posterior horn of the medial meniscus degeneration, Grade I (arrow). (**B**) Sagittal T1-weighted SPACE image of the right shoulder in a 20-year-old female badminton player: supraspinatus tendinopathy, Grade II (arrow). (**C**) Sagittal PD-FS image of the right knee in a 21-year-old male soccer player: posterior horn of the lateral meniscal lesion, Grade II (arrow). (**D**) Sagittal PD-FS image of the left ankle in a 19-year-old male badminton player: calcaneal bone marrow edema (arrow).

In the HPA group, shoulder lesions were exclusively confined to the supraspinatus tendon. Among the 103 asymptomatic shoulders, 7 lesions (6.8%) were detected, including 3 Grade-I and 4 Grade-II lesions. In the asymptomatic knees (*n* = 99), 24 lesions (24.2%) were detected; meniscal lesions accounted for the largest proportion (20/99, 20.2%), of which 16 (16/20, 80.0%) were Grade I, 4 (4/20, 20.0%) were Grade II, and none were Grade III. Other knee findings included ligament abnormalities (4 joints, 4.0%) and bone marrow edema (3 joints, 3.0%). Among the 101 ankle joints, 10 lesions (9.9%) were detected. Bone marrow edema was the most frequently observed finding (6 joints, 5.9%), followed by Achilles tendinopathy and ligament abnormalities (3 joints each, 3.0%) (Figure 2).

### 3.3. Intra-Individual Correlation of Joint Abnormalities

Among the 53 athletes, 11 (20.8%) presented with abnormalities in more than one joint, and the distribution of affected joints per participant differed significantly between the HPA and LPA groups (*p* < 0.001). The ICC for joint abnormalities across different joints within the same individual was 0.114 (95% CI: 0.025–0.242, *p* = 0.004). Pairwise Fisher’s exact tests showed associations between abnormalities of the left and right knee (OR = 10.4, 95% CI: 1.89–69.91, *p* = 0.002) and between the left and right shoulder (OR = 36.13, 95% CI: 1.41–2689.80, *p* = 0.010).

### 3.4. Factors Associated with Joint Abnormalities

A GEE logistic regression model was applied to all 666 joints from 137 participants (Supplementary Table S1). After adjusting for sex, age, BMI, and joint site, high physical activity was significantly associated with higher odds of joint abnormalities (adjusted OR = 5.23, 95% CI: 1.55–17.71, *p* = 0.008). BMI was positively associated with the odds of joint lesions (adjusted OR = 1.09 per kg/m^2^ increase, 95% CI: 1.03–1.15, *p* = 0.001). Regarding joint site, the odds of abnormality were higher for knee joints than for ankle joints (adjusted OR = 3.29, 95% CI: 1.44–7.51, *p* = 0.005).

In the HPA group, GEE analysis identified the knee joint site (adjusted OR = 3.01, 95% CI: 1.18–7.67, *p* = 0.021) and higher BMI (adjusted OR = 1.06, 95% CI: 1.00–1.11, *p* = 0.023) as independent factors associated with lesions. No statistically significant associations were observed for sport type (high-impact vs. low-impact), training duration, sex, age, or PARS-3 score (Table 4).

### 3.5. Anatomical Distribution of Meniscal Lesions in HPA Athletes

**Figure 3:**
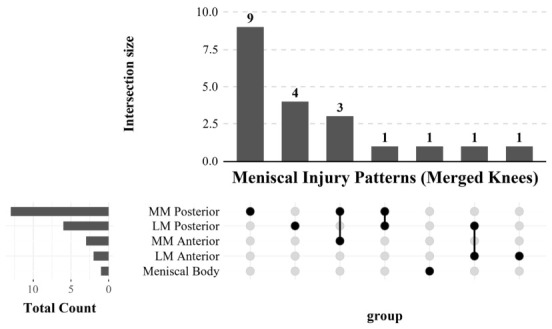
**UpSet plot of meniscal subregion involvement in HPA athletes (*n* = 20 knees).** The vertical bars represent the number of knees exhibiting a specific combination of lesions. The matrix of dots below the bars indicates the anatomical sites involved in each intersection: black dots mark regions with injury, gray dots mark regions without injury, and connected dots represent co-occurring abnormalities. The horizontal bars on the left show the total frequency of lesions for each anatomical subregion. MM: Medial meniscus; LM: lateral meniscus; HPA: high physical activity.

An intersection analysis of meniscal lesion distribution was performed on the 20 knees with meniscal lesions from 15 athletes (Figure 3). Lesions were most frequently located in the medial compartment (12/20, 60.0%) and in the posterior horn (13/20, 65.0%), and the posterior horn of the medial meniscus (MM Posterior) was the most frequently affected anatomical site (9/20, 45.0%). Of those studied, 7 knees (35.0%) showed involvement of more than one meniscal subregion. The most frequently observed multi-subregion combination was the concurrent injury of both the medial and lateral posterior horns (*n* = 4). In contrast, meniscal lesions in the LPA group were rare (*n* = 5), with no two knees sharing the same combination of subregional involvement.

## 4. Discussion

The study demonstrated a high prevalence of asymptomatic structural abnormalities in the knee, shoulder, and ankle joints of collegiate athletes compared with similarly aged LPA controls. These results suggest that asymptomatic joint pathologies are widespread among collegiate athletes and can develop silently prior to the onset of clinical symptoms, which is consistent with the findings of previous studies [23,24]. Moreover, more than one-fifth of affected athletes exhibited abnormalities in multiple joints simultaneously, indicating that joint abnormalities exhibit significant clustering within individual athletes. The bilateral co-occurrence of knee and shoulder abnormalities further supports non-independence and correlated injury risks across different joints within the same person.

The knee was the most frequently affected joint and the abnormalities were predominantly meniscal lesions, with athletes exhibiting a significantly higher prevalence of both Grade-I and Grade-II signal changes. This finding is consistent with previous studies reporting a high prevalence of meniscal lesions in the asymptomatic knee joints of both athletes and the general population [25,26].

Distinct lesion patterns were also observed in the shoulder and ankle. All shoulder joint pathologies in the HPA group were confined to supraspinatus tendon lesions, which are likely related to the cumulative stress of repetitive overhead arm movements [27]. Furthermore, the prevalence of bone marrow edema in the ankle joint was significantly higher than that observed in the LPA group. Notably, the LPA group demonstrated a near-zero prevalence of lesions in both the shoulder and ankle joints, highlighting that these pathologies are largely attributable to high-intensity athletic exposure rather than normal daily activities.

The intersection analysis indicated that the posterior horn of the meniscus is the most common location of asymptomatic lesions in the knee. The posterior horn of the medial meniscus is firmly attached to the joint capsule and tibia, resulting in limited mobility and rendering it highly susceptible to mechanical stress during torsion and load variation [28,29,30]. Since athletes frequently perform high-frequency, high-load maneuvers involving knee flexion, rotation, and landing, the medial meniscus is particularly vulnerable to microtrauma in this population. As meniscal lesions often do not elicit obvious clinical symptoms [31,32], they can develop silently, which explains the high detection rate observed in the asymptomatic knees in our study. In contrast, the prevalence of asymptomatic Achilles tendinopathy in our cohort was relatively low (3.0%), which differs from the higher incidence of Achilles pathology generally anticipated in athletic populations [33]. This lower prevalence may be attributed to several factors. Achilles tendinopathy shows a higher prevalence in older age [34], and our young collegiate athletes may not yet have accumulated sufficient loading exposure for structural tendon degeneration to develop. Unlike meniscal lesions, Achilles tendinopathy is frequently highly symptomatic—often presenting with localized pain or morning stiffness and excluded from the cohort [35,36], leaving only a small subset of truly asymptomatic structural variants. Furthermore, the HPA group comprised athletes from multiple disciplines, including low-impact sports such as swimming and shooting, which may have diluted the overall prevalence of Achilles tendinopathy compared with cohorts composed predominantly of runners or jumping athletes.

In the HPA group, neither sport type nor training duration was independently associated with joint lesions. A post hoc power analysis indicated that the statistical power was limited and the study may have been underpowered to detect subgroup differences. Consequently, the results should be interpreted cautiously and require further investigations with larger samples.

Our findings demonstrated that high-intensity training was significantly associated with the risk of asymptomatic pathologies in weight-bearing joints. Although these joints present no clinical symptoms, signal changes and structural irregularities are already detectable via high-resolution MRI sequences. These “silent” pathological changes may represent the phase of overt injuries under sustained high-intensity loading [37]. MRI can be used to sensitively identify asymptomatic structural abnormalities, including bone marrow edema and meniscal and ligamentous lesions. Systematic MRI-based surveillance may therefore represent a promising strategy for early identification of subclinical joint pathology, with the potential to support injury prevention and prolong athletes’ competitive careers [38,39,40].

To our knowledge, this study is among the first to incorporate a similarly aged control group of less-active young adults, addressing a notable gap in the literature by isolating the effects of athletic exposure on joint pathology. Furthermore, unlike previous studies that focused predominantly on single joints, our study provided a comprehensive multi-joint assessment (knee, shoulder, and ankle) within the same cohort, offering a more holistic view of the musculoskeletal burden imposed by professional training. Finally, the high-resolution MRI protocol utilized in our study offered superior sensitivity for detecting subtle structural abnormalities compared with conventional two-dimensional sequences.

However, the cross-sectional design prevents us from establishing a causal timeline, and long-term longitudinal follow-up is necessary to determine the prognostic value of these MRI findings. The non-random selection of joints in the LPA group may have introduced selection bias and may have led to underestimation of true abnormality prevalence in shoulder and ankle joints. Additionally, the study population was limited to collegiate athletes, so the results may not be generalizable to older professional athletes or pediatric populations. Self-reported PARS-3 scores may be subject to recall bias and may not capture the full complexity of training load. Future studies should incorporate objective monitoring tools, such as wearable sensors or GPS-based workload tracking, to provide a more accurate quantification of training exposure and its relationship to joint health.

Looking ahead, the findings of this study open several avenues for future research. Longitudinal studies are needed to determine which asymptomatic lesions progress to symptomatic injury and which remain clinically silent. Integrating objective workload metrics and imaging biomarkers from high-resolution MRI may also help identify thresholds of cumulative stress that predispose athletes to structural joint changes. Ultimately, these insights could inform the development of targeted preventive strategies, including individualized training modifications, early detection protocols, and sport-specific conditioning programs aimed at mitigating joint degeneration before symptoms arise.

## 5. Conclusions

Asymptomatic structural abnormalities are substantially prevalent in collegiate athletes and exhibit significant intra-individual clustering, particularly affecting the knee meniscus, shoulder supraspinatus tendon, and ankle bone marrow. These subclinical lesions develop silently prior to the onset of clinical symptoms and are associated with high-intensity athletic loading. Systematic MRI-based surveillance may represent a promising strategy for early risk identification in athletes and individuals with high levels of habitual physical activity.

## Figures and Tables

**Table 1:** MRI scanning protocol.

Joints	Sequence	TR (ms)/TE (ms)	FOV (mm)	Slices	Matrix	Slice Thickness (mm)	Flip Angle (deg)	TA (min:s)
**Knee**	Sagittal 3D T1-weighted SPACE	700/10	170 × 170	144	256 × 256	0.66	120	5:38
	Sagittal 3D PD-weighted FS SPACE	1200/16	170 × 170	144	256 × 256	0.66	120	5:29
**Shoulder**	Coronal 3D T1-weighted SPACE	700/21	200 × 200	72	256 × 256	0.78	120	5:49
	Coronal 3D PD-weighted FS SPACE	1200/22	200 × 200	72	256 × 256	0.78	120	5:12
**Ankle**	Sagittal 3D T1-weighted SPACE	700/21	162 × 200	88	256 × 208	0.78	120	4:21
	Sagittal 3D PD-weighted FS SPACE	1200/24	162 × 200	88	256 × 208	0.78	120	4:24

Note: TR = Repetition time; TE = echo time; TA = total acquisition time; 3D = three-dimensional; PD = proton density; FS = fat-suppressed.

**Table 2:** Characteristics of enrolled participants.

Characteristics	Total (*n* = 137)	HPA Group (*n* = 53)	LPA Group (*n* = 84)	*p*-Value
Age (years)	21.0 (20.0, 23.0)	20.0 (19.0, 21.0)	22.0 (20.0, 23.0)	<0.001
BMI (kg/m^2^)	22.0 (20.4, 24.0)	22.1 (20.8, 24.1)	21.8 (19.8, 24.0)	0.319
Height (cm)	169.0 (162.0, 177.0)	176.0 (170.0, 181.0)	165.0 (161.0, 170.3)	<0.001
Weight (kg)	63.0 (56.0, 72.0)	70.0 (61.0, 78.0)	60.0 (53.8, 68.0)	<0.001
Male sex, *n* (%)	58 (42.3%)	37 (69.8%)	21 (25.0%)	<0.001
PARS-3 score	24.0 (6.0, 64.0)	64.0 (60.0, 64.0)	8.0 (4.0, 18.0)	<0.001
Participants with abnormalities, *n* (%)	31 (22.6%)	26 (49.1%)	5 (6.0%)	<0.001

Note: Continuous variables are presented as median (interquartile range, IQR) and categorical variables are presented as frequencies (percentages). HPA: high physical activity; LPA: low physical activity; BMI: body mass index; PARS-3: Physical Activity Rating Scale-3.

**Table 3:** Prevalence of asymptomatic MRI abnormalities in HPA and LPA groups.

Joint/Lesion Type	HPA Group, Joints (*n* = 303)			LPA Group, Joints (*n* = 363)			*p*-Value
	Left	Right	Total *n* (%)	Left	Right	Total *n* (%)	
**Shoulder joint**	52	51	103	47	49	96	
Any lesion present	3	4	7 (6.8%)	0	0	0 (0.0%)	0.014
Supraspinatus tendon injury	3	4	7 (6.8%)	0	0	0 (0.0%)	0.014
Grade I	2	1	3 (2.9%)	0	0	0 (0.0%)	0.248
Grade II	1	3	4 (3.9%)	0	0	0 (0.0%)	0.123
**Knee joint**	50	49	99	75	76	151	
Any lesion present	13	11	24 (24.2%)	3	4	7 (4.6%)	<0.001
Ligament lesion	3	1	4 (4.0%)	0	0	0 (0.0%)	0.203
Cartilage lesion	0	1	1 (1.0%)	0	1	1 (0.7%)	1.000
Bone marrow edema	3	0	3 (3.0%)	1	0	1 (0.7%)	0.301
Meniscal lesion	10	10	20 (20.2%)	2	3	5 (3.3%)	<0.001
Grade I	9	7	16 (16.2%)	2	3	5 (3.3%)	0.001
Grade II	1	3	4 (4.0%)	0	0	0 (0.0%)	0.023
**Ankle joint**	51	50	101	58	58	116	
Any lesion present	3	7	10 (9.9%)	0	1	1 (0.9%)	0.003
Bone marrow edema	2	4	6 (5.9%)	0	1	1 (0.9%)	0.039
Ligament lesion	0	3	3 (3.0%)	0	0	0 (0.0%)	0.100
Achilles tendinopathy	1	2	3 (3.0%)	0	0	0 (0.0%)	0.100

Note: Data are presented as *n* (%). Percentages in the “Total *n* (%)” columns are calculated based on the total number of evaluated joints of the corresponding joint type within each activity group. A single joint may present with more than one type of lesion. HPA: high physical activity; LPA: low physical activity.

**Table 4:** Prevalence and GEE analysis of factors associated with joint lesions in the HPA group.

Variable	Total Joints, *n*	Joints with Lesions, *n* (%)	Adjusted OR	95% CI	*p*-Value
**Sport Type**					
Low-impact (Ref)	63	8 (12.7%)	1.00	—	—
High-impact	240	33 (13.8%)	1.51	0.56–4.07	0.412
**Training Duration**					
≤5 years (Ref)	147	18 (12.3%)	1.00	—	—
5–10 years	129	22 (17.1%)	1.71	0.63–4.61	0.280
>10 years	27	1 (3.7%)	0.31	0.04–2.53	0.258
**Sex**					
Female (Ref)	93	12 (12.9%)	1.00	—	—
Male	210	29 (13.8%)	1.36	0.52–3.61	0.531
**Joint Location**					
Ankle (Ref)	101	10 (9.9%)	1.00	—	—
Knee	99	24 (24.2%)	3.01	1.18–7.67	0.021
Shoulder	103	7 (6.8%)	0.68	0.19–2.42	0.548
**BMI**	303	—	1.06	1.00–1.11	0.023
**Age**	303	—	1.02	0.79–1.32	0.883
**PARS-3 score**	303	—	1.01	0.98–1.04	0.443

Note: Data are presented as number of joints with lesions (percentage of total screened joints in that group, e.g., 103 for HPA shoulders). Odds ratios (ORs) and 95% confidence intervals (CIs) were estimated using generalized estimating equations (GEEs) with a logit link and binomial distribution. Ref: Reference category.

## Data Availability

The original contributions presented in this study are included in the article. Further inquiries can be directed to the corresponding authors.

## Supplementary Materials

The following supporting information can be downloaded at https://www.mdpi.com/article/10.3390/diagnostics16091335/s1, Table S1: GEE logistic regression analysis of factors associated with joint abnormalities in the overall cohort.

## Abbreviations

The following abbreviations are used in this manuscript:

MRI	Magnetic Resonance Imaging
BMI	Body Mass Index
PARS-3	Physical Activity Rating Scale-3
HPA	High Physical Activity
LPA	Low Physical Activity
CI	Confidence Interval
OR	Odds Ratio
GEE	Generalized Estimating Equations
IOC	International Olympic Committee

## References

[1] Liaghat B., Pedersen J.R., Young J., Thorlund J., Juul-Kristensen B., Juhl C. (2021). Joint Hypermobility in Athletes Is Associated with Shoulder Injuries: A Systematic Review and Meta-Analysis. BMC Musculoskelet. Disord..

[2] Prieto-González P., Martínez-Castillo J.L., Fernández-Galván L.M., Casado A., Soporki S., Sánchez-Infante J. (2021). Epidemiology of Sports-Related Injuries and Associated Risk Factors in Adolescent Athletes: An Injury Surveillance. Int. J. Environ. Res. Public Health.

[3] Del Re A., Alexandrov A. (2023). Epidemiology of Sports Injuries. Translational Sports Medicine.

[4] Muthuri S.G., McWilliams D.F., Doherty M., Zhang W. (2011). History of Knee Injuries and Knee Osteoarthritis: A Meta-Analysis of Observational Studies. Osteoarthr. Cartil..

[5] Palmer D., Cooper D., Whittaker J.L., Emery C., Batt M.E., Engebretsen L., Schamasch P., Shroff M., Soligard T., Steffen K. (2022). Prevalence of and Factors Associated with Osteoarthritis and Pain in Retired Olympians Compared with the General Population: Part 2—The Spine and Upper Limb. Br. J. Sports Med..

[6] Møller M., Isaksen Johansen S., Myklebust G., Nielsen R.O., Möller S., Mikkelsen U., Wedderkopp N., Lind M. (2024). Health Problems and Injury Management in Adolescent Handball: The Safeplay One-Season Cohort Study of 679 Players. Br. J. Sports Med..

[7] Beletsky A., Okoroha K.R., Cabarcas B., Garcia G.H., Gowd A.K., Meyer J., Vadhera A.S., Singh H., Gursoy S., White G.M. (2022). Magnetic Resonance Imaging Findings of the Asymptomatic Shoulder May Impact Performance, Not Future Injury List Placement in Major League Baseball Pitchers. Arthrosc. Sports Med. Rehabil..

[8] Bezuglov E.N., Khaitin V.Y., Lyubushkina A.V., Lazarev A.M., Gorinov A.V., Sivakova E.Y., Rumiantseva E.I., Lychagin A.V. (2020). The Effect of Training Experience and Leg Dominance on the Prevalence of Asymptomatic Intraarticular Changes of the Knee Joints in Adult Professional Male Soccer Players. Sports Med. Open.

[9] Chang X.-D., Yang P., Mu X.-Y., Ma W.-L., Zhou M. (2018). Evaluation of Knees in Asymptomatic Amateur Ice Hockey Players Using 3.0-T Magnetic Resonance Imaging: A Case-Control Study. Chin. Med. J..

[10] López-Valenciano A., Ruiz-Pérez I., Garcia-Gómez A., Vera-Garcia F.J., De Ste Croix M., Myer G.D., Ayala F. (2020). Epidemiology of Injuries in Professional Football: A Systematic Review and Meta-Analysis. Br. J. Sports Med..

[11] Lin C.-I., Mayer F., Wippert P.-M. (2022). The Prevalence of Chronic Ankle Instability in Basketball Athletes: A Cross-Sectional Study. BMC Sports Sci. Med. Rehabil..

[12] Zhou X., Imai K., Liu X.-X., Chen Z., Watanabe E., Zeng H. (2025). Epidemiological Characteristics of Injury in 7–22-Year-Old Badminton Players by Age and Sex. Sci. Rep..

[13] Ishikawa H., Cushman D.M., Tashjian R.Z., Chalmers P.N. (2025). Examining the Prevalence of Sports-Related Injuries in Collegiate Baseball Players. Orthop. J. Sports Med..

[14] Liang D.Q., Liu S.J. (1994). The Relationship between Stress Level and Physical Exercise for College Students. Chin. Ment. Health J..

[15] Bahr R., Clarsen B., Derman W., Dvorak J., Emery C.A., Finch C.F., Hägglund M., Junge A., Kemp S., International Olympic Committee Injury and Illness Epidemiology Consensus Group (2020). International Olympic Committee Consensus Statement: Methods for Recording and Reporting of Epidemiological Data on Injury and Illness in Sports 2020 (Including the STROBE Extension for Sports Injury and Illness Surveillance (STROBE-SIIS)). Orthop. J. Sports Med..

[16] Zanetti M., Bruder E., Romero J., Hodler J. (2000). Bone Marrow Edema Pattern in Osteoarthritic Knees: Correlation between MR Imaging and Histologic Findings. Radiology.

[17] Faruch-Bilfeld M., Lapegue F., Chiavassa H., Sans N. (2016). Imaging of Meniscus and Ligament Injuries of the Knee. Diagn. Interv. Imaging.

[18] Peterfy C.G., Guermazi A., Zaim S., Tirman P.F.J., Miaux Y., White D., Kothari M., Lu Y., Fye K., Zhao S. (2004). Whole-Organ Magnetic Resonance Imaging Score (WORMS) of the Knee in Osteoarthritis. Osteoarthr. Cartil..

[19] Szaro P., Nilsson-Helander K., Carmont M. (2021). MRI of the Achilles Tendon—A Comprehensive Pictorial Review. Part One. Eur. J. Radiol. Open.

[20] Zlatkin M.B., Iannotti J.P., Roberts M.C., Esterhai J.L., Dalinka M.K., Kressel H.Y., Schwartz J.S., Lenkinski R.E. (1989). Rotator Cuff Tears: Diagnostic Performance of MR Imaging. Radiology.

[21] Stoller D.W., Martin C., Crues J.V., Kaplan L., Mink J.H. (1987). Meniscal Tears: Pathologic Correlation with MR Imaging. Radiology.

[22] Yoshida K., Yoshimura I., Otsuka N., Masuda K., Nakamura N., Shigemori Y. (2026). Effects of Physical Activities during Senior High School on Bone Mineral Density in University Freshmen. Sport Sci. Health.

[23] Bezuglov E.N., Lyubushkina A.V., Khaitin V.Y., Tokareva A.V., Goncharov E.N., Gorinov A.V., Sivakova E.Y., Sereda A.P. (2019). Prevalence of Asymptomatic Intra-Articular Changes of the Knee in Adult Professional Soccer Players. Orthop. J. Sports Med..

[24] Sanders S., Ibounig T., Haas R., Jones M., Rämö L., Docking S., Järvinen T., Taimela S., Hoffmann T., Buchbinder R. (2025). Rotator Cuff Imaging Abnormalities in Asymptomatic Shoulders: A Systematic Review. J. Orthop. Sports Phys. Ther..

[25] Culvenor A.G., Øiestad B.E., Hart H.F., Stefanik J.J., Guermazi A., Crossley K.M. (2019). Prevalence of Knee Osteoarthritis Features on Magnetic Resonance Imaging in Asymptomatic Uninjured Adults: A Systematic Review and Meta-Analysis. Br. J. Sports Med..

[26] Beals C.T., Magnussen R.A., Graham W.C., Flanigan D.C. (2016). The Prevalence of Meniscal Pathology in Asymptomatic Athletes. Sports Med..

[27] Connor P.M., Banks D.M., Tyson A.B., Coumas J.S., D’Alessandro D.F. (2003). Magnetic Resonance Imaging of the Asymptomatic Shoulder of Overhead Athletes: A 5-Year Follow-up Study. Am. J. Sports Med..

[28] DePhillipo N.N., Moatshe G., Chahla J., Aman Z.S., Storaci H.W., Morris E.R., Robbins C.M., Engebretsen L., LaPrade R.F. (2018). Quantitative and Qualitative Assessment of the Posterior Medial Meniscus Anatomy: Defining Meniscal Ramp Lesions. Am. J. Sports Med..

[29] Masouros S.D., McDermott I.D., Amis A.A., Bull A.M.J. (2008). Biomechanics of the Meniscus-Meniscal Ligament Construct of the Knee. Knee Surg. Sports Traumatol. Arthrosc..

[30] Daszkiewicz K., Łuczkiewicz P. (2026). Biomechanics of Medial Meniscus Tears in the Context of Pain: A Finite Element Analysis. Front. Bioeng. Biotechnol..

[31] Tsai L., Matzkin E., Jones M.H., Miller R.E., Katz J.N. (2025). Potential Sources of Pain in Symptomatic Degenerative Meniscal Tear: A Narrative Review. Osteoarthr. Cartil. Open.

[32] Englund M., Guermazi A., Gale D., Hunter D.J., Aliabadi P., Clancy M., Felson D.T. (2008). Incidental Meniscal Findings on Knee MRI in Middle-Aged and Elderly Persons. N. Engl. J. Med..

[33] Florit D., Pedret C., Casals M., Malliaras P., Sugimoto D., Rodas G. (2019). Incidence of Tendinopathy in Team Sports in a Multidisciplinary Sports Club over 8 Seasons. J. Sports Sci. Med..

[34] Moonot P., Dakhode S. (2024). Current Concept Review of Achilles Tendinopathy. J. Clin. Orthop. Trauma.

[35] de Vos R.-J., van der Vlist A.C., Zwerver J., Meuffels D.E., Smithuis F., van Ingen R., van der Giesen F., Visser E., Balemans A., Pols M. (2021). Dutch Multidisciplinary Guideline on Achilles Tendinopathy. Br. J. Sports Med..

[36] Scott A., Squier K., Alfredson H., Bahr R., Cook J.L., Coombes B., de Vos R.-J., Fu S.N., Grimaldi A., Lewis J.S. (2020). ICON 2019: International Scientific Tendinopathy Symposium Consensus: Clinical Terminology. Br. J. Sports Med..

[37] Bezuglov E., Khaitin V., Lazarev A., Brodskaia A., Lyubushkina A., Kubacheva K., Waskiewicz Z., Petrov A., Maffulli N. (2021). Asymptomatic Foot and Ankle Abnormalities in Elite Professional Soccer Players. Orthop. J. Sports Med..

[38] Torvaldsson K., Fagher K., Derman W., Engebretsen L., Lindblom H., Lopes A.D., Runciman P., Schwellnus M., Soligard T., Sonesson S. (2025). Injury and Illness Epidemiology in Elite Athletes during the Olympic, Youth Olympic and Paralympic Games: A Systematic Review and Meta-Analysis. Br. J. Sports Med..

[39] Jimenez C., Verhagen E. (2025). Reimagining Athlete Monitoring for True Indicative Injury Prevention. BMJ Open Sport Exerc. Med..

[40] White T., Alway P., Brooke-Wavell K., Wedatilake T., King M., Peirce N. (2025). MRI Screening for Lumbar Bone Stress Injuries in Young Male Cricket Fast Bowlers: A 15-Year Retrospective Cohort Study. Br. J. Sports Med..

