# Supplementary material for: Asymptomatic Abnormalities in the Knee, Shoulder, and Ankle Joints of Collegiate Athletes: A Cross-Sectional MRI-Based Comparative Study
Source: Diagnostics (Basel). 2026 Apr 29;16(9):1335. doi: 10.3390/diagnostics16091335 (PMC13163245; doi:10.3390/diagnostics16091335)
Supplement: Supplementary file 1 [file diagnostics-16-01335-s001.zip › diagnostics-4276126-supplementary.pdf]

Table S1: GEE Logistic Regression Analysis of Factors Associated with Joint Abnormalities in the Overall Cohort

Variable	Participants, n	Total joints, n	Lesions, n (%)	Adjusted OR	95% CI	p
**Group**						
LPA (ref.)	84	363	8 (2.2%)	1.000	—	—
HPA	53	303	41 (13.5%)	5.233	1.546–17.713	0.008
**Sex**						
Female (ref.)	79	—	—	1.000	—	—
Male	58	—	—	1.055	0.458–2.433	0.899
**Age** (per year), mean ± SD	21.2 ± 1.9	—	—	0.817	0.659–1.014	0.066
**BMI** (per kg/m^2), mean ± SD	22.5 ± 3.7	—	—	1.089	1.034–1.146	0.001
**Joint site**						
Ankle (ref.)	—	217	11 (5.1%)	1.000	—	—
Knee	—	250	31 (12.4%)	3.286	1.438–7.510	0.005
Shoulder	—	199	7 (3.5%)	0.607	0.178–2.073	0.426
**Overall**	137	666	49 (7.4%)	—	—	—

Note — OR = odds ratio; CI = confidence interval; HPA = high physical activity; LPA = low physical activity; BMI = Body Mass Index; ref.= reference category; SD = standard deviation. The GEE logistic regression model was fitted with a binomial distribution and exchangeable correlation structure (α = 0.112), accounting for within-participant clustering of multiple joints per individual. Age and BMI are continuous variables reported as mean ± SD at the participant level.
